# Supplementary material for: Mutations in PCYT2 disrupt etherlipid biosynthesis and cause a complex hereditary spastic paraplegia
Source: Brain. 2019 Oct 22;142(11):3382–97. doi: 10.1093/brain/awz291 (PMC6821184; doi:10.1093/brain/awz291)
Supplement: awz291_Supplementary_Data [file awz291_supplementary_data.zip › awz291-Suppl_data/Supplementary_Data4.zip!/Supplementary information 3 - fibroblast lipidomics.zip!/Fibroblast lipidomics/Patient1 vs Patient2/Statistical plots and data/TG[O] species/Heatmap.pdf]

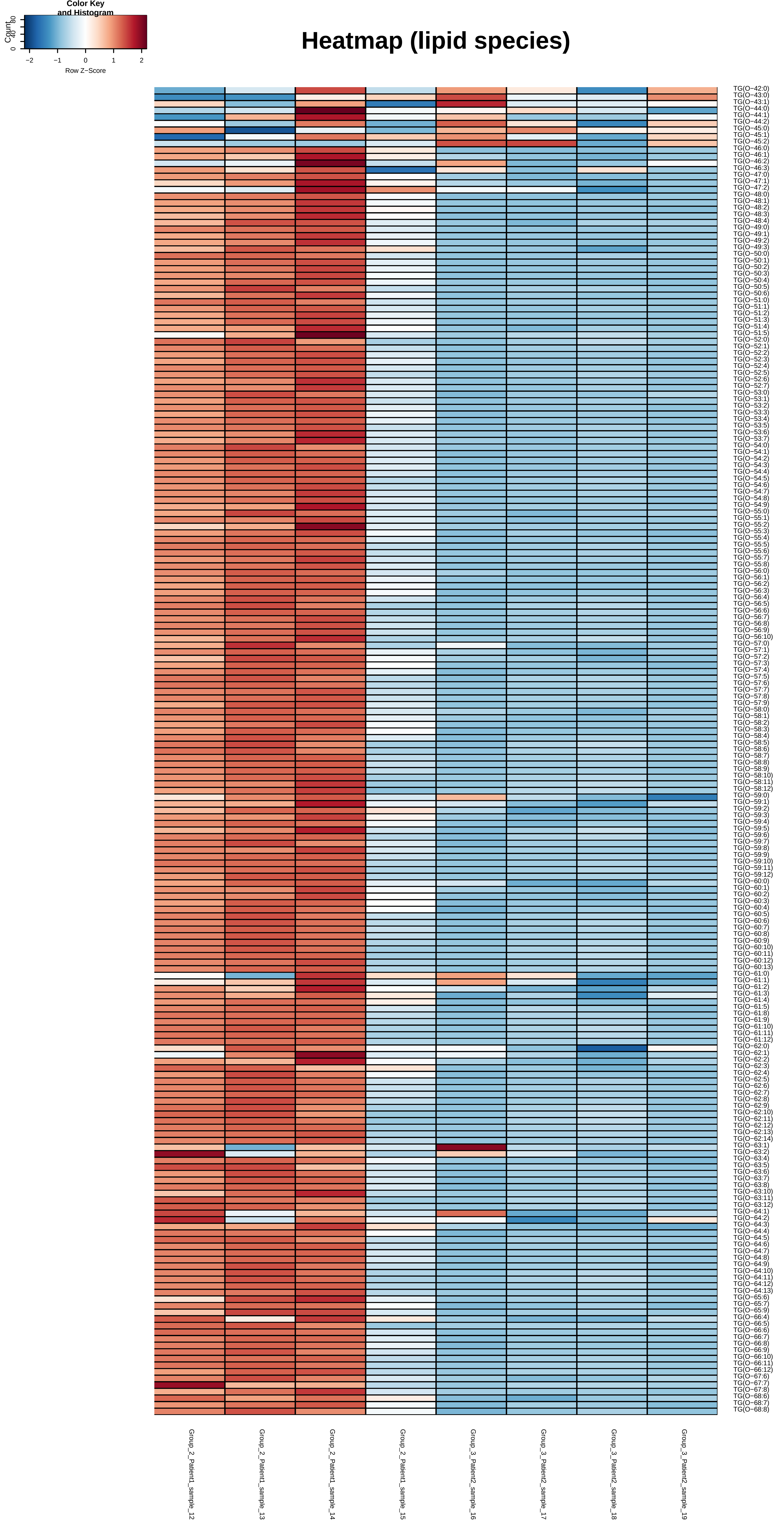
Color Key
and Histogram
Count
0 40 80
−2 −1 0 1 2
Row Z−Score
Heatmap (lipid species)
TG(O−42:0)
TG(O−43:0)
TG(O−43:1)
TG(O−44:0)
TG(O−44:1)
TG(O−44:2)
TG(O−45:0)
TG(O−45:1)
TG(O−45:2)
TG(O−46:0)
TG(O−46:1)
TG(O−46:2)
TG(O−46:3)
TG(O−47:0)
TG(O−47:1)
TG(O−47:2)
TG(O−48:0)
TG(O−48:1)
TG(O−48:2)
TG(O−48:3)
TG(O−48:4)
TG(O−49:0)
TG(O−49:1)
TG(O−49:2)
TG(O−49:3)
TG(O−50:0)
TG(O−50:1)
TG(O−50:2)
TG(O−50:3)
TG(O−50:4)
TG(O−50:5)
TG(O−50:6)
TG(O−51:0)
TG(O−51:1)
TG(O−51:2)
TG(O−51:3)
TG(O−51:4)
TG(O−51:5)
TG(O−52:0)
TG(O−52:1)
TG(O−52:2)
TG(O−52:3)
TG(O−52:4)
TG(O−52:5)
TG(O−52:6)
TG(O−52:7)
TG(O−53:0)
TG(O−53:1)
TG(O−53:2)
TG(O−53:3)
TG(O−53:4)
TG(O−53:5)
TG(O−53:6)
TG(O−53:7)
TG(O−54:0)
TG(O−54:1)
TG(O−54:2)
TG(O−54:3)
TG(O−54:4)
TG(O−54:5)
TG(O−54:6)
TG(O−54:7)
TG(O−54:8)
TG(O−54:9)
TG(O−55:0)
TG(O−55:1)
TG(O−55:2)
TG(O−55:3)
TG(O−55:4)
TG(O−55:5)
TG(O−55:6)
TG(O−55:7)
TG(O−55:8)
TG(O−56:0)
TG(O−56:1)
TG(O−56:2)
TG(O−56:3)
TG(O−56:4)
TG(O−56:5)
TG(O−56:6)
TG(O−56:7)
TG(O−56:8)
TG(O−56:9)
TG(O−56:10)
TG(O−57:0)
TG(O−57:1)
TG(O−57:2)
TG(O−57:3)
TG(O−57:4)
TG(O−57:5)
TG(O−57:6)
TG(O−57:7)
TG(O−57:8)
TG(O−57:9)
TG(O−58:0)
TG(O−58:1)
TG(O−58:2)
TG(O−58:3)
TG(O−58:4)
TG(O−58:5)
TG(O−58:6)
TG(O−58:7)
TG(O−58:8)
TG(O−58:9)
TG(O−58:10)
TG(O−58:11)
TG(O−58:12)
TG(O−59:0)
TG(O−59:1)
TG(O−59:2)
TG(O−59:3)
TG(O−59:4)
TG(O−59:5)
TG(O−59:6)
TG(O−59:7)
TG(O−59:8)
TG(O−59:9)
TG(O−59:10)
TG(O−59:11)
TG(O−59:12)
TG(O−60:0)
TG(O−60:1)
TG(O−60:2)
TG(O−60:3)
TG(O−60:4)
TG(O−60:5)
TG(O−60:6)
TG(O−60:7)
TG(O−60:8)
TG(O−60:9)
TG(O−60:10)
TG(O−60:11)
TG(O−60:12)
TG(O−60:13)
TG(O−61:0)
TG(O−61:1)
TG(O−61:2)
TG(O−61:3)
TG(O−61:4)
TG(O−61:5)
TG(O−61:8)
TG(O−61:9)
TG(O−61:10)
TG(O−61:11)
TG(O−61:12)
TG(O−62:0)
TG(O−62:1)
TG(O−62:2)
TG(O−62:3)
TG(O−62:4)
TG(O−62:5)
TG(O−62:6)
TG(O−62:7)
TG(O−62:8)
TG(O−62:9)
TG(O−62:10)
TG(O−62:11)
TG(O−62:12)
TG(O−62:13)
TG(O−62:14)
TG(O−63:1)
TG(O−63:2)
TG(O−63:4)
TG(O−63:5)
TG(O−63:6)
TG(O−63:7)
TG(O−63:8)
TG(O−63:10)
TG(O−63:11)
TG(O−63:12)
TG(O−64:1)
TG(O−64:2)
TG(O−64:3)
TG(O−64:4)
TG(O−64:5)
TG(O−64:6)
TG(O−64:7)
TG(O−64:8)
TG(O−64:9)
TG(O−64:10)
TG(O−64:11)
TG(O−64:12)
TG(O−64:13)
TG(O−65:6)
TG(O−65:7)
TG(O−65:9)
TG(O−66:4)
TG(O−66:5)
TG(O−66:6)
TG(O−66:7)
TG(O−66:8)
TG(O−66:9)
TG(O−66:10)
TG(O−66:11)
TG(O−66:12)
TG(O−67:6)
TG(O−67:7)
TG(O−67:8)
TG(O−68:6)
TG(O−68:7)
TG(O−68:8)
Group_2_Patient1_sample_12
Group_2_Patient1_sample_13
Group_2_Patient1_sample_14
Group_2_Patient1_sample_15
Group_3_Patient2_sample_16
Group_3_Patient2_sample_17
Group_3_Patient2_sample_18
Group_3_Patient2_sample_19
